# Supplementary material for: Complexome profiling of the Chlamydomonas psb28 mutant reveals TEF5 as an early PSII assembly factor
Source: Plant Cell. 2025 Mar 17;37(6):koaf055. doi: 10.1093/plcell/koaf055 (PMC12164590; doi:10.1093/plcell/koaf055)
Supplement: koaf055_Supplementary_Data [file koaf055_supplementary_data.zip › psb28_interactive.zip!/plots/intensity/qualityPlots/peptides/SABC_Cre06.g273750.t1.1.html]

### Cre06.g273750.t1.1

Trivial name: SABC  
SeqCoverage [median]: 42.00  
Mass: 48.15 kDa  
  
 

| Sequence | MissedCleavage | Mass [Da] | wtIntensity | muIntensity | Score |
| --- | --- | --- | --- | --- | --- |
| AASIDKPVVYTPR | 1 | 1415.78 | 4379.59 | 10881.49 | 107.37 |
| ALASNPR | 0 | 727.40 | 0.00 | 1809.82 | 104.11 |
| DQDEREYNLVVGSR | 1 | 1678.79 | 5373.12 | 10659.66 | 121.24 |
| FVGETNVVPATSLLAK | 0 | 1644.91 | 8494.58 | 17003.01 | 167.73 |
| GLVEQQGSPTEIIK | 0 | 1497.80 | 6062.55 | 19386.53 | 170.68 |
| GLVEQQGSPTEIIKRPR | 2 | 1907.06 | 0.00 | 2075.54 | 63.04 |
| KLNIDHDKR | 2 | 1137.63 | 0.00 | 1445.33 | 63.08 |
| LLLLDEPFGALDAVVR | 0 | 1739.98 | 1212.92 | 0.00 | 234.79 |
| LLLLDEPFGALDAVVRK | 1 | 1868.08 | 1431.77 | 0.00 | 95.65 |
| TMMGFNASDVDSTPIV | 0 | 1683.75 | 0.00 | 1202.35 | 103.08 |
| TPFIMK | 0 | 735.40 | 0.00 | 2479.40 | 107.65 |
| TSVMFRPHDIK | 1 | 1329.69 | 1259.91 | 6686.73 | 94.19 |
| TVAENIK | 0 | 773.43 | 0.00 | 4278.21 | 92.47 |
| TVPPESGEGALTTVGANVADK | 0 | 2012.01 | 0.00 | 552.14 | 30.97 |
| VVVFNR | 0 | 732.43 | 0.00 | 1245.75 | 78.00 |
|  |  |  | 28214.4 | 79706.0 |  |
